# Noonan Syndrome Type 5 Diagnosed by Next-Generation Sequencing: A Report of a Rare Pediatric Case

**DOI:** 10.7759/cureus.115089

**Published:** 2026-08-24

**Authors:** Sanae Kheir, Jihane Ahmidi, Mariam Tajir, Maria Rkain, Abdeladim Babakhouya

**Affiliations:** 1 Pediatrics, Centre Hospitalier Universitaire Mohammed VI, Oujda, MAR; 2 Medical Genetics, Centre Hospitalier Universitaire Mohammed VI, Oujda, MAR; 3 Medical Genetics, Mohammed I University, Oujda, MAR; 4 Pediatrics, Faculty of Medicine and Pharmacy of Oujda, Mohammed I University, Oujda, MAR; 5 Pediatric Gastroenterology, Centre Hospitalier Universitaire Mohammed VI, Oujda, MAR

**Keywords:** hypertrophic cardiomyopathy (hcm), next-generation sequencing (ngs), noonan syndrome, raf1, rasopathy

## Abstract

Noonan syndrome (NS) is a genetically heterogeneous RASopathy characterized by distinctive facial features, growth impairment, developmental delay, and congenital heart disease. Variants in the *RAF1* gene are strongly associated with hypertrophic cardiomyopathy (HCM). We report the case of a seven-year-old girl presenting with HCM, growth retardation, developmental delay, and characteristic dysmorphic features. Clinical suspicion of NS was raised during infancy. Initial investigations, including urinary glycosaminoglycan analysis and targeted molecular testing of the *PTPN11* gene, were negative. Owing to persistent clinical suspicion, next-generation sequencing (NGS) was subsequently performed and identified a heterozygous pathogenic *RAF1* variant, c.770C>T (p.Ser257Leu), establishing the diagnosis of NS type 5. This case highlights the importance of expanded molecular testing, particularly NGS, in patients with suspected RASopathies and reinforces the association between the RAF1 p.Ser257Leu variant and HCM.

## Introduction

Noonan syndrome (NS) (OMIM 163950) is an eponymous disorder first described by the pediatric cardiologist Jacqueline Noonan in 1968 [1]. It is an autosomal dominant RASopathy with an estimated prevalence ranging from 1 in 1,000 to 1 in 2,500 live births [1,2]. It is characterized by marked clinical variability, including facial dysmorphism, short stature, skeletal abnormalities, developmental disorders, and cardiovascular involvement [3,4]. Mutations in the *RAF1* gene account for approximately 5-10% of NS cases [1,5] and are particularly associated with hypertrophic cardiomyopathy (HCM), which represents one of the major causes of morbidity in affected patients [6,7]. We report the case of a patient with NS type 5, confirmed by next-generation sequencing (NGS) after several years of clinical evolution and following negative initial targeted molecular testing.

## Case presentation

**Figure 1:**
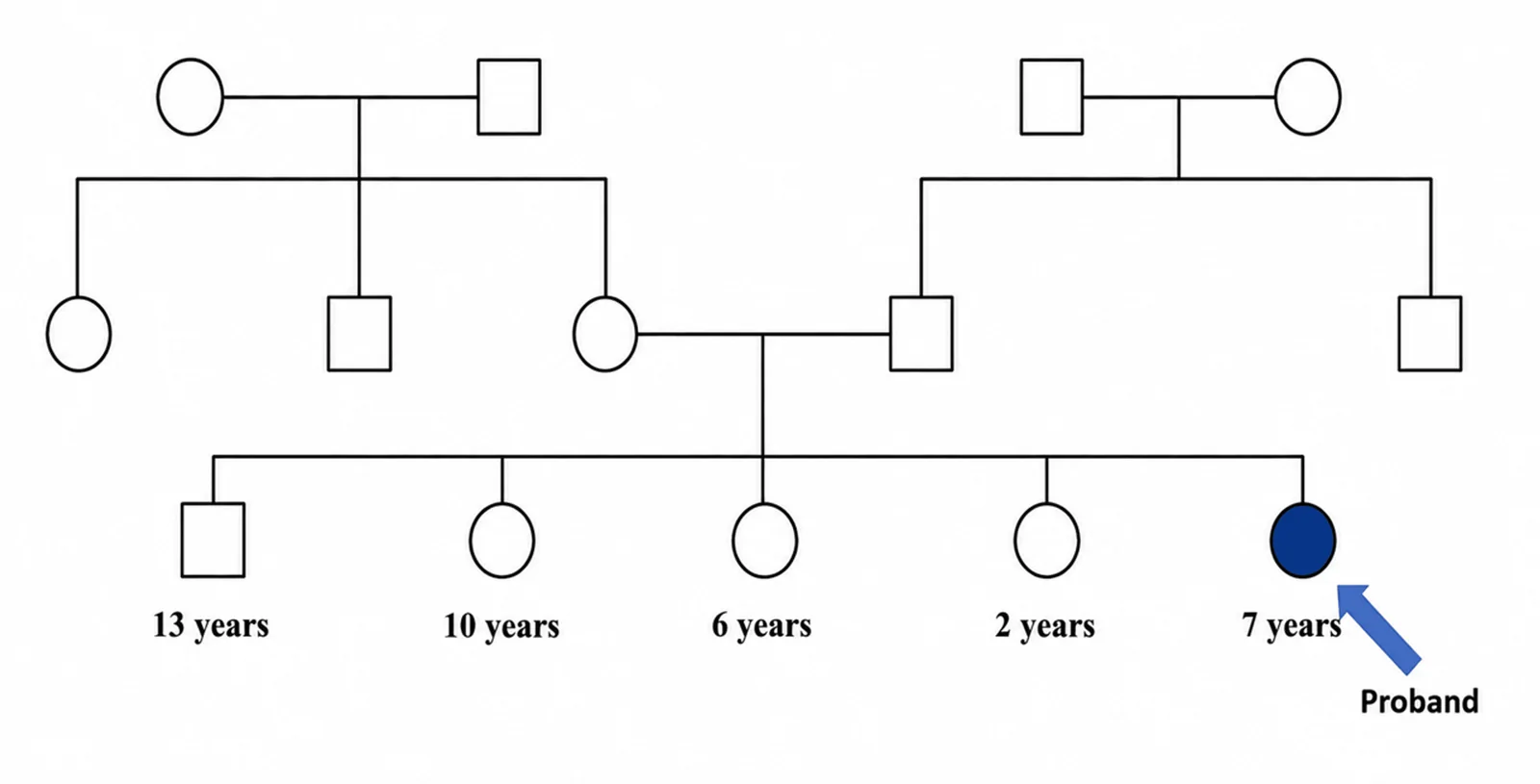
Pedigree of the family affected by Noonan syndrome. The arrow indicates the proband. Numbers below each symbol represent the age (in years) of the family members.

A seven-year-old girl, born to non-consanguineous parents, with no significant family history, was evaluated (Figure 1).

The patient’s medical history dates back to the age of 10 months, when HCM was diagnosed in the context of respiratory distress and failure to thrive. During follow-up, the child exhibited persistent growth retardation as well as delayed psychomotor development.

**Figure 2:**
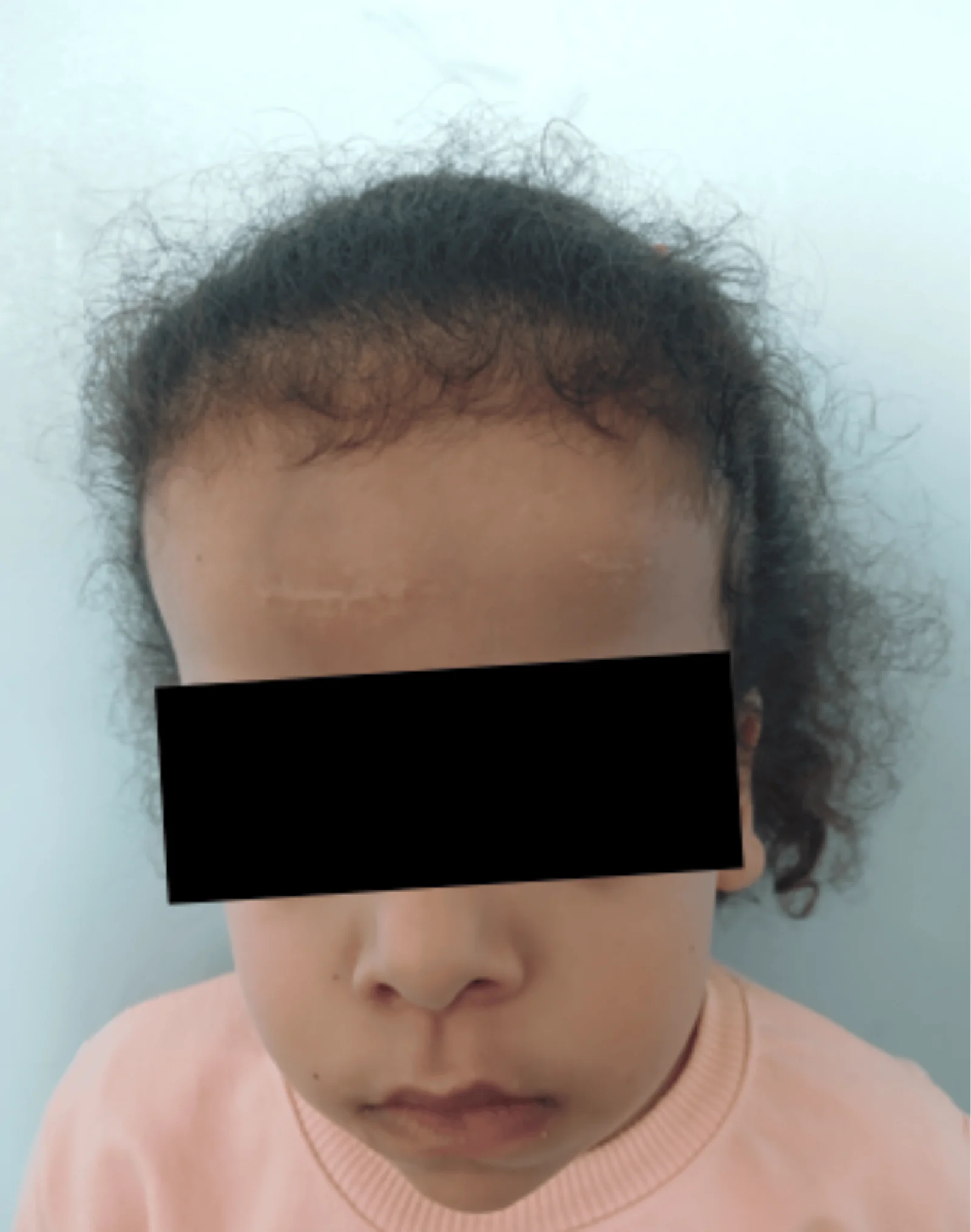
Frontal view of the patient demonstrating facial dysmorphic features.

Clinical examination revealed a dysmorphic syndrome characterized by a glabellar hemangioma, a broad and prominent forehead, downward- and outward-slanting palpebral fissures, a depressed nasal bridge with anteverted nostrils, a prominent philtrum, a pronounced Cupid’s bow, low-set protruding earlobes, and a short neck (Figure 2). A narrow chest with pectus excavatum, padded hands with deep palmar creases, and a broad great toe were also noted.

**Figure 3:**
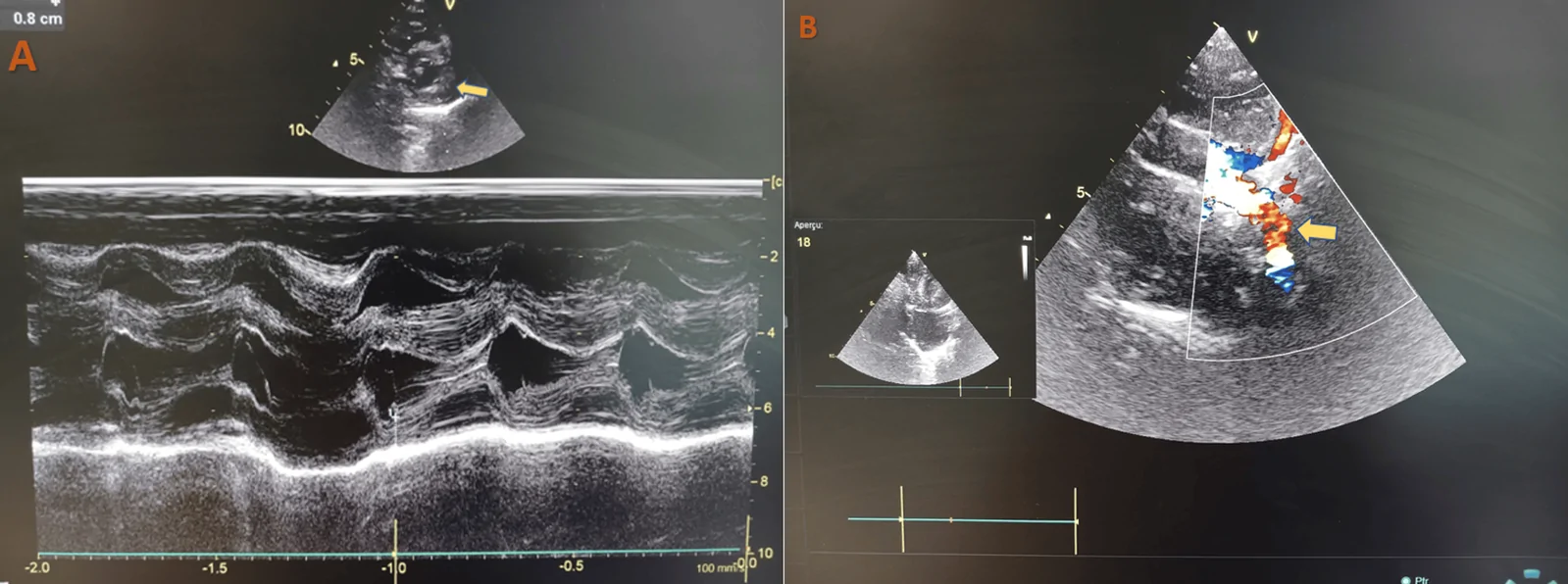
Transthoracic echocardiographic findings in a patient with RAF1-associated Noonan syndrome type 5. (A) M-mode image showing left ventricular hypertrophy (arrow). (B) Color Doppler image demonstrating mitral regurgitation (arrow).

Echocardiography revealed left ventricular hypertrophy associated with thickening and structural abnormalities of the mitral valve, mitral valve prolapse, and mitral regurgitation resulting in dilation of the left heart chambers (Figure 3).

Given the association of HCM with dysmorphic features, several syndromic conditions were considered in the differential diagnosis, particularly mucopolysaccharidosis and NS. Mucopolysaccharidosis was initially considered because of these clinical features; however, urinary glycosaminoglycan analysis was negative, arguing against this diagnosis. An initial targeted molecular analysis of the *PTPN11* gene, using polymerase chain reaction amplification followed by sequencing of exons 2, 3, 4, 7, 8, 12, and 13, did not identify any pathogenic variant.

Owing to the strong clinical suspicion, NGS was performed and identified a heterozygous pathogenic variant in the *RAF1* gene, c.770C>T (p.Ser257Leu), confirming the diagnosis of NS type 5 (OMIM 611553). Parental testing was not performed, and the inheritance status of the *RAF1* variant remains unknown. The patient remains under regular pediatric cardiology follow-up, with stable cardiac status at the latest evaluation.

## Discussion

NS exhibits significant genetic heterogeneity involving several genes of the RAS/MAPK signaling pathway [1,3]. This diversity explains the diagnostic challenges encountered in some patients when targeted molecular analyses yield negative results, highlighting the importance of integrating clinical features with molecular investigations in the diagnostic workup of NS [4].

In 2007, activating mutations of the *RAF1* gene were identified as a cause of NS and are strongly associated with HCM [5,6]. Of these, p.Ser257Leu is one of the most frequently reported and well-characterized *RAF1* variants [7,8]. In studies of genotype-phenotype correlation, this mutation has been associated with a high prevalence of HCM, often early in onset and occasionally severe [7]. Similar findings have been reported in patients carrying *RAF1* mutations, in whom HCM may be associated with severe cardiac outcomes [9].

The clinical features observed in our patient are consistent with those previously reported in the literature, including typical facial dysmorphism, growth retardation, pectus excavatum, and progressive HCM. HCM is recognized as one of the most common and severe cardiovascular manifestations in patients with RASopathies and may show progressive worsening during childhood [10]. Recent reports have expanded the spectrum of cardiac manifestations associated with *RAF1* mutations, highlighting the phenotypic heterogeneity of NS [11]. The mitral valve involvement observed in our patient has also been described in patients carrying the *RAF1* c.770C>T (p.Ser257Leu) variant [7].

This case also emphasizes the significance of NGS for the diagnostic workup of RASopathies. Despite strong clinical suspicion, targeted analysis of the *PTPN11* gene was negative. NGS can identify pathogenic variants, including those in *RAF1*, and provide molecular confirmation of diagnosis [3,12]. This approach is very effective in increasing the diagnostic yield in genetically heterogeneous diseases such as NS [12].

A limitation of this report is that it describes a single patient, and no additional cases with the same *RAF1* variant were available for comparison. Nevertheless, this case adds to the existing clinical literature on *RAF1*-associated NS and highlights the diagnostic value of NGS.

## Conclusions

NS type 5 should be suspected in children presenting with characteristic facial dysmorphism associated with HCM and developmental delay. This case is consistent with the strong genotype-phenotype correlation between the *RAF1* p.Ser257Leu variant and cardiac involvement, especially HCM. It further emphasizes the importance of comprehensive molecular testing, including NGS, for achieving an early and accurate diagnosis of genetically heterogeneous RASopathies. Early recognition may allow appropriate multidisciplinary management and long-term follow-up. In patients with *RAF1*-associated NS, regular cardiological surveillance is particularly important because of the strong association with HCM. In our patient, continued multidisciplinary follow-up, particularly cardiological monitoring, is important to assess the evolution of HCM and guide management.
